# Suicide prevention by emergency nurses: perceived self-efficacy in assessment, management and referral at Kenyatta National Hospital in Kenya

**DOI:** 10.1186/s12991-019-0240-8

**Published:** 2019-08-27

**Authors:** Rachel Maina, David Bukusi, Manasi Kumar

**Affiliations:** 1 Clinical Psychologist University of Nairobi, 10834-00400, Nairobi, Kenya; 2 Tilburg University, Tilburg, The Netherlands; 3 Head of VCT and HIV Prevention/Youth Center KNH, Nairobi, Kenya; 4 Department of Psychiatry, University of Nairobi, Nairobi, Kenya

**Keywords:** Suicide risk assessment, Suicide self-efficacy, Accident and Emergency Nurses, Suicide risk management, Suicide risk referral

## Abstract

**Background:**

Emergency Departments are underutilized settings for suicide prevention and management as patients with occult (camouflaged) suicides and suicidal ideation are rarely screened by nurses and other health workers in these sites. The under-detection rates could be a result of lack of suicide assessment and management confidence among the hospital staff. The aim of the study was to find out the perceived self-efficacy in suicide risk assessment, management and referral among nurses working in an emergency department within a lower income country.

**Method:**

The Risk Assessment and Management Self-Efficacy Scale (RAMSES) was administered among nurses in an emergency department (ED) within an urban region in a descriptive study. The risk assessment, management and referral domains among 64 respondents were evaluated using mean and standard deviation calculations in SPSS v 21.

**Results:**

The total RAMSES composite score in risk assessment, management and referral was 6.19 (SD 2.107) with risk assessment having the lowest mean score of 6.09 (SD 2.08), while risk referral process mean score was the highest at 6.55 (SD 2.36). The nurses had the least confidence in developing a written risk management plan 5.68 (SD 2.51) as well as using screening instruments to assess risk 5.90 (SD 2.15).

**Findings:**

Nurses in emergency department have below average self-efficacy in suicide assessment and management necessitating training as well as integration of protocols that could enhance effective utilization of emergency departments as suicide prevention and management settings.

## Introduction

Globally, one in five people who have committed suicide have had contact with a health professional [12]. Suicidal assessment and management on initial contact with a health professional is an important preventive and curative intervention; yet in many settings, most cases go undetected. In America, around 1089 people who had visited hospitals including emergency departments in between 2010 and 2014 committed suicide and this number is said to be a small proportion of the actual population [19]. This is higher compared to suicides committed in medical settings. In 2014 and 2015, 16 and 30 inpatients suicides were reported, respectively, though these data are marred by incomplete data [21]. The suicides rates were reported through National Violent Death Reporting System (NVDRS) which collects these data from forty states in USA, as well as, Puerto Rico and District of Columbia. In Sub-Saharan Africa, there is a dearth of such data. However, a study done in Cameroon showed that 24% of suicide victims had sought medical care prior to the completed suicide, yet no mental health care was given, and above this, 87% of nurses lacked depression screening knowledge yet suicide is a key depression symptom [10].

The emergency department has been shown to have the potential to identify suicidal cases in several settings [15], mainly because suicidal behavior requires urgent management. A study conducted among African Americans showed that out of all the patients presenting at the Emergency Department with suicidal symptoms, only 25% of them were identified as having suicidal ideations with 76% of the respondents being discharged home and only 39% of them having at least one follow up session [9]. Another study has shown that the more frequent the emergency department visits, the higher the risk of suicide [11]. The study did not identify the reasons for this trend though extrapolated that number of hospital visits is an independent factor in suicide risk.

While most of the patients seeking services at the Kenyatta National Hospital in Kenya go through the Accident and Emergency (A and E) department before being referred for other medical services, the A and E department is often underutilized as a site for suicide prevention and management. Evidently, 8–12% of patients with occult suicide present in emergency settings with other comorbid critical ailments such as stroke, chronic obstructive pulmonary disease and heart attacks [6, 20].

Some of the factors which could lead to low detection of suicidal cases include lack of training among key personnel and work pressure at the A and E [3, 10]. Studies have shown that nurses in the emergency department are often uncomfortable attending to patients who had deliberately tried to harm themselves [17]. These nurses often lack sufficient skills in attending to such patients [5, 15, 17]. In a study done by Rutto et al. [17] in Kenyatta National Hospital A and E Department, a third of the nurses felt uncomfortable and nervous when attending to patients, with more than half of them expressing frustration when treating them as is the case among nurses in similar departments worldwide. A study conducted across United States in seven states by Betz et al. [4] showed that 64–70% of the nurses lacked confidence in suicide risk assessment skills; while 46–56% lacked counseling skills which are important in treating patients with suicidal tendencies as biomedical model is not enough in management. It is, therefore, vital to have nurses who are skilled and confident in assessing and managing suicide.

The lack of suicide-specific intervention skills could account for the uncomfortable feelings nurses have around patients with suicidal tendencies. However, there is a paucity of data to ascertain this outcome. In their American study, Betz et al. [4] found out that the odds of self-efficacious Emergency Department nurses screening for suicidal symptoms was 1.60 meaning nurses who were confident in suicide screening were more likely to screen most or all patients for suicidal symptoms than nurses who were not confident in the same [4].

**Fig. 1:**
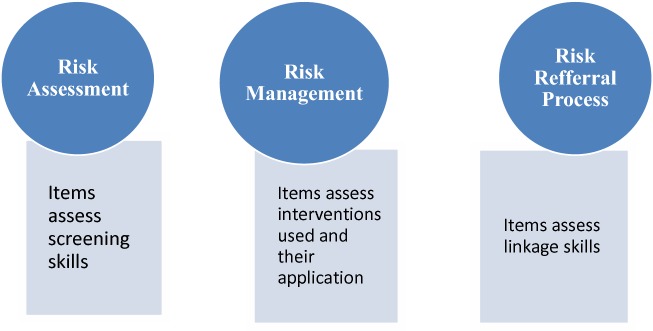
Risk assessment and management self-efficacy scale (RAMSES) domains

The main aim of this study was to assess the self-efficacy of nurses in suicide risk assessment and management in A and E setting. In this study we asked A and E nurses in Kenya’s Kenyatta National Hospital whether they were confident in assessing, managing and referring patients with suicide risk (Fig. 1). To answer this question, we administered the Risk Assessment and Management Self-Efficacy Scale (RAMSES) in a descriptive study.

## Methods

### Study design

This was a descriptive study conducted in Kenyatta National Hospital’s Accident and Emergency Department among nurses who are in direct contact with patients.

### Sampling

Purposive sampling was done to select only the nurses who were directly involved in patient care. This included nurse trainees and nurses employed by the hospital who were approached during their morning reports meeting and invited to participate in the study.

### Subjects

There were a total of 64 nurses who consented to participate in the study. The recruitment stopped at this number as no more nurses consented to participate in the study. The nurses work in the hospital either as employed staff or trainee nurses. Whichever criteria they fitted in, they had to be in direct contact with patients as ascertained by a verbal self-report during the recruitment part of the study.

### Consenting and data collection procedure

Eligible and consenting participants completed a demographic survey that collected information on social and demographic characteristics. After giving an informed consent, the nurses were requested to fill in the socio-demographic questionnaire and the Risk Assessment and Management Self-Efficacy Scale (RAMSES) questionnaire. The consenting and data collection was done at the same time. Surveys were conducted by a research assistant and took approximately 20 min per nurse. It took a week to complete data collection.

### Tool

The self-efficacy variable was collected using Risk Assessment and Management Self-Efficacy Scale (RAMSES) questionnaire. The questionnaire consists of 18 items that assess training programs associated with risk management. The tool has been developed utilizing Albert Bandura’s Social Cognitive theory and has been found to have robust psychometric properties (with validity *r* = 0.71 and reliability of Cronbach’s *α* of 0.96 showing high internal consistency [7]. RAMSES contains the following three domains; assessment, management and referral. It is a self-administered tool with respondents being prompted to assess their self perceived efficacy levels in a Likert scale that ranges from 0 for no confidence to 10 for complete confidence [7]. A composite score is then generated after adding all the items in RAMSES and dividing the result by 18.

### Ethical approval

The study was given ethical approval by the Kenyatta National Hospital and University of Nairobi Ethics Review Committee. Approval was also given by the Accident and Emergency Department as well as Research and Programs Department within the hospital.

### Analysis

The data were analyzed using SPSS version 20 where distribution tables were used to show the percentages, frequencies, standard deviation and mean distribution of the variables. Significant correlation between perceived self efficacy, measured as an ordinal variable, and demographic variables, measured as categorical and ordinal variables, was analyzed using Fischer’s Exact Test because the cell counts were below five as some expected counts were small.

## Results

Nurses who agreed to participate were 64 in total with 57.8% of them being females, while a third (30.2%) of them had an income of above Kshs. 40,000. More than half (65.6%) of them had undergraduate education and 55.6% of them were unemployed; while, 3.2% were self-employed probably representing the nurse trainees attached to KNH. One participant did not indicate their gender (Table 1). We did not detect any significant correlation between socio-demographic variables and total self-efficacy composite score.

**Table 1:** Demographic characteristics of nurses at Kenyatta National Hospital’s Accident and Emergency Department

Demographic information	N (%)	Total self-efficacy composite score Fischer’s exact test–P value (set alpha level of 0.05)
Gender		
Female	37 (57.8)	0.766
Male	26 (40.6)	
Unknown (missing)	1 (1.6)	
Age (years)		
≤ 25	33 (58.9)	0.109
≥ 26	23 (41.1)	
Highest level of education		
Secondary	15 (24.2)	0.438
College Diploma	3 (4.8)	
Undergraduate	42 (67.7)	
Masters	2 (3.2)	
Occupation		
Unemployed	35 (55.6)	0.438
Employed	26 (41.3)	
Self-employed	2 (3.2)	
Income		
0–9999	36 (57.1)	0.5
10,000–19,999	1 (1.6)	
20,000–29,999	1 (1.6)	
30,000–39,999	6 (9.5)	
40,000 and above	19 (30.2)	

RAMSES has not been previously used in the study setting, and hence, internal consistency was calculated; though this was not part of what the study set out to do. Correlation calculations were done between the mean scores of the domains and in relation to the total mean composite score. Significant correlations were found between the domains mean score and total mean composite score (Domain A *r* = 0.916, *p *= 0.000; Domain B *r* = 0.923, *p *= 0.000; Domain C *r* = 0.875, *p* = 0.000). This implied that the internal consistency of RAMSES was maintained in the Kenyan cultural context where it was being applied.

The mean scores of the perceived suicide risk assessment self-efficacy in domains A1–A6 ranged from 5.90 (SD 2.15) to 6.55 (SD 2.07) with a total composite score of 6.09 (SD 2.08) (see Table 2). These scores were slightly lower than perceived self-efficacy in risk referral process score of domain C whose mean ranged from 6.56 (SD 2.15) to 6.74 (SD 2.55) in domains C1–C4 with a total composite score of 6.55 (SD 2.36). Due to these low scores, the total composite score in risk assessment, management and referral was low at 6.19 (SD 2.107).

**Table 2:** Suicide self-efficacy domain-based outcomes (mean and SD) among nurses working in KNH A and E (*N* = 64)

Suicide item domain	Suicide item description	Mean (SD)
How confident are you that you can		
A1	Interview people to elicit key information about risk factors	6.32 (2.21)
A2	Use screening instrument to assess risk	5.90 (2.15)
A3	Identify a person who is presenting risk to self	6.52 (2.01)
A4	Identify a person who is presenting risk to others	6.55 (2.07)
A5	Differentiate between people presenting high risk and low risk	6.00 (2.15)
A6	Synthesize relevant information in a formal or written risk assessment	6.13 (2.26)
Domain A total composite score-risk assessment		6.09 (2.08)
B1	Use specific interventions focusing on risk of self-harm or self-neglect	6.13 (2.06)
B2	Help people to minimize the severity of risk to self	6.66 (1.98)
B3	Use specific interventions focusing on risks of harm to (or neglect of) others	6.26 (2.07)
B4	Help people to minimize the severity of risk to others	6.84 (2.26)
B5	Develop rapport with people who present significant risks	7.16 (2.05)
B6	Manage risks in line with organizational confidentiality policies	6.34 (2.38)
B7	Use strategies to avoid malpractices liability or disciplinary action	6.16 (2.30)
B8	Develop a formal or written risk management plan	5.68 (2.51)
Domain B total-risk management		6.25 (2.27)
C1	Appropriately judge whether or not a person should be referred to an external service or professional on the basis of risk	6.69 (2.13)
C2	Identify an appropriate service to refer someone on the basis of risk	6.56 (2.15)
C3	Successful refer and engage a person with an appropriate service	6.60 (2.16)
C4	Motivate a person to successfully self-refer to an appropriate service	6.74 (2.55)
Domain C total-risk referral process		6.55 (2.36)
Total self-efficacy in suicide risk assessment management and referral		6.19 (2.11)

## Discussion

The mean perceived total self-efficacy for suicide assessment and management was below the upper quartile as none of the individual items scores were > 7. Unexpectedly, despite the nurses having a very low mean score in suicide risk assessment, they had a slightly higher mean score in risk management and even higher in risk referral process. This showed a diverse level of confidence which is not dependent on whether the initial factors that should be considered when doing suicide assessment and management are self-efficacious. Moreover, this could be because they may have higher competency in detection of other risk behaviors or other components of referral process. It could also be that if any suicidal symptom was detected using any means, the nurses integrated suicide management practices and referred patient for further management. This is evidenced in a qualitative study done among the same population where if a patient is found to be suicidal, they are referred for counseling [13]. The respondents in this study had a high index of suicide risk suspicion for patients who were raped, between ages 20 and 35 and who have an alcohol problem [13]. The level of confidence in risk referral process was quite high in this study compared to the other two domains considering there are no formal referral protocols in this particular setting. However, compared to a study in India, another setting that did not have risk assessment and management protocols as well, this level of confidence and hands-on functioning is reflective of a setting with no policies or protocols for referral process [8].

Majority of the respondents in our study had lower self-efficacy than of another study in India that found 73% respondents have above-average mean scores (> 7) [8]. This inverse comparison is also seen in domain scores where respondents in the Indian study showed high scores in risk assessment and lowest in risk referral which is inversely proportional to our study findings. The Indian study results are replicated in a study in Tunisia among primary care physicians where respondents were more confident in detecting suicide (54%) than in treating people with issues related to suicide (23.4%) [18]. This may be due to perceived complexity in managing suicide [18].

Using formal assessment and management structures was scored quite low as nurses showed very low self-efficacy in using risk screening instruments (A2) and even lower in coming up with a write-up of a formal management plan (B8). This means that nurses are less likely to use suicide screening instruments; a situation that a study in United States has established reduces the chances of health workers in emergency departments screening for suicidal symptoms (OR 1.60) [2]. This underutilizes usage of emergency departments as suicide prevention sites as patients will most likely not tell if not asked. The lack of suicide assessment protocols in Kenyatta National Hospital’s emergency department further aggravates this outcome [13]. This leads to management of physiological symptoms such as stroke, chronic obstructive pulmonary disease and heart attacks; while 8–12% of occult suicides go undetected and unmanaged [6, 20]. Moreover, people with other mental health complications that co-occur with suicide may go undiagnosed. This includes people with Major Depressive Disorder who have been found in Kenya to be 19 times more likely to have suicidal thoughts [16]. This is among the reasons why suicide is amid the priority conditions identified in the Mental Health Gap Action Programme as in need of an intervention [22]. Suicide screening has been shown to increase identification of occult suicides [1], decrease rate of suicide attempts and this rate tends to decrease significantly once an intervention that includes a safety plan is integrated [14]. The results of these study show that this is less likely to happen. However, this trajectory may change with training as studies have shown that training in use of brief suicide screening tools increases chances of Emergency Department nurses screening for suicide from 36 to 95% (*p* < 0.001) [1, 2].

Forming rapport with patients seems not to be a problem among nurses working in emergency departments most likely because it is part of routine care for all forms of health management. However, the emergency department remains a fast paced unit where work flow, stress and training gaps may impend efficient use of rapport in assessment and management of patients at risk of suicide.

## Conclusion

The study findings show that Nurses could benefit from trainings on suicide assessment and management, and especially in using suicide screening tools that can be administered within a short time, bearing in mind that this is a busy hospital setting, and that can reflect a true diagnostic picture, owing to validity and reliability of the tool. Further research may need to be done on suicide assessment and management trainings that would effectively be integrated in such a busy setting. The Nurses could also benefit from formal protocols and policies in assessment and management of patients to have a smooth transition in management of patients with suicide risk. These measures could ensure that Emergency Departments are effectively utilized as sites for suicide prevention and management.

## Limitation

The study was held in a hospital and in the institutions emergency department; hence, results are not generalizable to the community and other departments within the hospital. However, since the hospital is likely to have a higher prevalence of people with suicidal ideations, the study’s preferential setting is ideal. Moreover, the nurses at the emergency department are most likely among the first in a hospital setting to get in contact with patients who have suicidal ideation. Therefore, this study is significant for the hospital emergency departments.

## Data Availability

All the data collected and analyzed in this study are with the corresponding author and may be provided once requested.
